# Anxiolytic-Like Effect of a Salmon Phospholipopeptidic Complex Composed of Polyunsaturated Fatty Acids and Bioactive Peptides

**DOI:** 10.3390/md11114294

**Published:** 2013-10-30

**Authors:** Nabila Belhaj, Frédéric Desor, Céline Gleizes, Frédéric M. Denis, Elmira Arab-Tehrany, Rachid Soulimani, Michel Linder

**Affiliations:** 1 Laboratory of Biomolecular Engineering, University of Lorraine, École Nationale Supérieure d’Agronomie et des Industries Alimentaires (ENSAIA), National School on Agronomy and Food Industry 2, Avenue de la Forêt de Haye TSA 40602 54518, Vandoeuvre Cedex, France; E-Mails: belhaj.nabila@hotmail.fr (N.B.); gleizes.celine@gmail.com (C.G.); elmira.arab-tehrany@univ-lorraine.fr (E.A.-T.); 2 Food Neurotoxicology, Micropollutants and Food Contaminants (MRCA), University of Lorraine, Research Unit on Animal and Functionality of Animal Products (URAFPA), Institut National de la Recherche Agronomique (INRA), BP 4102, Metz 57040, France; E-Mails: frederic.desor@univ-lorraine.fr (F.D.); rachid.soulimani@univ-lorraine.fr (R.S.); 3 Le Stum Laboratory, Larmor Plage 56260, France; E-Mail: infomed@labo-lestum.com

**Keywords:** nanoemulsion, anti-anxiety activity, PUFA, reactive oxygen species, neuroprotective effect

## Abstract

A phospholipopeptidic complex obtained by the enzymatic hydrolysis of salmon heads in green conditions; exert anxiolytic-like effects in a time and dose-dependent manner, with no affection of locomotor activity. This study focused on the physico-chemical properties of the lipidic and peptidic fractions from this natural product. The characterization of mineral composition, amino acid and fatty acids was carried out. Stability of nanoemulsions allowed us to realize a behavioral study conducted with four different tests on 80 mice. This work highlighted the dose dependent effects of the natural complex and its various fractions over a period of 14 days compared to a conventional anxiolytic. The intracellular redox status of neural cells was evaluated in order to determine the free radicals scavenging potential of these products in the central nervous system (CNS), after mice sacrifice. The complex peptidic fraction showed a strong scavenging property and similar results were found for the complex as well as its lipidic fraction. For the first time, the results of this study showed the anxiolytic-like and neuroprotective properties of a phospholipopeptidic complex extracted from salmon head. The applications on anxiety disorders might be relevant, depending on the doses, the fraction used and the chronicity of the supplementation.

## 1. Introduction

Anxiety is defined as a complex emotional state, a combination of feelings of fear and worry, often accompanied by instability or tension, associated with an apprehensive anticipation of a probable future threat [1,2]. Its acute physiological effects include breathing difficulties, an increase in blood pressure, palpitations, hyperthermia, dizziness and nausea [3]. Although anxiety is a normal and adaptive natural human emotion, higher levels coupled with persistence may cause abnormal behaviors and become pathological [4,5]. Anxiety disorders are one of the most common classes of mental disorders. The lifetime prevalence of these troubles was estimated to be 5.1 and 2 percent in US and Europe, respectively [6,7]. Furthermore, they are involved in numerous other psychiatric disorders, such as depression, generalized anxiety disorders, phobias, panic attacks, obsessive-compulsive disorder or post-traumatic stress disorders [5].

Human studies have revealed that peripheral oxidative stress is associated with high anxiety levels and other psychiatric troubles including pathological anxiety e.g., obsessive-compulsive disorder and panic disorder [8,9,10]. In addition, several studies conducted on rodent models have also demonstrated a close link between high anxiety-related behavior and central as well as peripheral oxidative stress [10,11,12,13]. Oxidative stress occurs from the imbalance between reactive oxygen species (ROS) production and the cellular antioxidant defense system in favor of the former [14]. The production of excess ROS is detrimental to the cell, inducing damages in lipids membrane, carbohydrates, proteins and nucleic acids. Because of its higher oxygen requirement, poor antioxidant defenses and its lipid-rich constitution, the brain is considered particularly susceptible to oxidative stress [15,16]. At last, the detrimental consequences of oxidative stress in this organ include mitochondrial dysfunction, altered neuronal signaling and inhibition of neurogenesis, resulting in an overall brain activity alteration [10,11]. Anxiety is usually treated with medication including benzodiazepines or selective serotonin reuptake inhibitors. Although showing good responses against this pathology, the use of such drugs may be unsafe because of their adverse effects, encompassing excessive sedation, disinhibition, psychosis, paranoia as well as dependence inducing properties [17,18,19]. Therefore, alternative compounds, mainly issued from natural resources, are evaluated for their anxiolytic potential.

Fish is a source of bioactive and functional molecules with materials including polyunsaturated fatty acids (PUFAs), peptides, polysaccharides, minerals, vitamins, antioxidants and enzymes. These materials are used as food ingredients, nutraceuticals and pharmaceuticals [20]. Marine food production generates a large amount of by-products, which have low commercial value, but significant economic and environmental benefits [21,22]. Several studies have reported that bioactive peptides can be obtained from enzymatic proteolysis of different marine bio-resources, including fish by-products [23], and the scavenging property of such marine peptides against free radicals have been demonstrated [24,25]. In addition, enzymatic extraction from fish by-products allows for obtaining high quality fish oil [26,27], that contains PUFAs, particularly eicosapentaenoic acid (EPA, C20:5 *n*-3) and docosahexaenoic acid (DHA, C22:6 *n*-3). Health benefits of these long chain PUFAs (LC-PUFAs) have been proven for humans [28], especially in the treatment of mood disorders [29] as well as anxiety troubles [30].

The present study was designed firstly to characterize the physico-chemical properties of a natural phospholipopeptidic complex obtained from the enzymatic hydrolysis of salmon heads.

Vectorization of LC-PUFAs by marine phospholipids is increasingly studied due to its applications in nutrition and health fields. The high stability of these molecules depends on their composition and interactions with associated antioxidant molecules [31].

However, the influence of polar lipids rich in EPA and DHA esterified in sn-2 position, associated with peptides from marine origin has not been studied. Because this complex was found particularly rich in peptides and PUFAs, this characterization was also extended to its peptidic and lipidic fractions.

In a second time, behavioral investigations including the follow-up on levels of anxiety and locomotor activity were performed in adult male mice, subsequently to acute and sub-acute (14 days) dietary supplements, for the three treatments studied at a single and double dose. Finally, the intracellular redox status of mice’ neural cells was assessed *ex vivo* after a two week period of supplementation.

## 2. Results

### 2.1. Phospholipopeptidic Complex

The phospholipopeptidic complex was obtained by enzymatic hydrolysis, performed in a stirred batch thermostated reactor using the pH-stat method. The degree of hydrolysis was about 11.5% to obtain a high percentage of peptides with molecular weight ranging between 4200 and 13,200 Da [32]. It appears that a low degree of hydrolysis improves emulsion capacity, emulsion stability and fat absorption. The protease used and the degree of hydrolysis could greatly affect the functional properties of resulting hydrolysate [33]. Indeed, a hydrolysis degree between 8 and 11 led to the production of small peptides [34]. Increasing the degree of hydrolysis generated many low molecular weight peptides, which improved the radical scavenging ability of salmon muscle hydrolysate [35]. Lipidic and peptidic fractions and minerals were characterized separately in this study.

#### 2.1.1. Characterization of Lipids

Salmon head lipids were composed of 65% polar lipids and 35% triacylglycerols. Polar lipids were constituted by 28.11% of phosphatidylcholine (PC), 13.53% of phosphatidylethanolamine (PE), 9.80% of phosphatidylserine (PS) and 6.24% of phosphatidylinositol (PI) (results not shown). The results obtained by Iatroscan analysis are in accordance with the study of Miller *et al.* [36], where PC and PE made up the majority of the phospholipid fraction in Atlantic salmon [37].

**Table 1:** Minerals, fatty acids and amino acids composition of phospholipopeptidic complex obtained from salmon head.

Minerals				Fatty acids		Amino acids				
(μg/g)				(%)			nmole/mg	%	g/kg	%
Component	PLPC	Component	PLPC	Fatty acids	Percentage	Amino acids	PLPC			Salmon marine collagen peptide *
Arsenic	<1.1	Rubidium	0.547 ± 0.05	C14:0	3.54 ± 0.01	Asparagine	606.49	11.44	80.12	-
Barium	<2	Antimony	<0.1	C15:0	0.24 ± 0.02	Glutamic acid	713.83	13.47	105.02	12.22
Beryllium	<0.45	Samarium	<0.01	C15:1	0.27 ± 0.01	Serine	290.26	5.48	30.50	4.23
Bismuth	<0.09	Tin	<0.3	C16:0	17.04 ± 0.04	Glycine	822.50	15.52	61.74	23.77
Cadmium	<0.1	Strontium	34.21 ± 3.42	C16:1 *n*-7	0.31 ± 0.01	Histidine	nq	nq	nq	1.61
Cerium	<0.12	Tantalum	<0.01	C16:1 *n*-9	4.10 ± 0.05	Arginine	369.15	6.96	64.30	6.08
Cobalt	<0.2	Terbium	<0.005	C16:3 *n*-3	0.42 ± 0.02	Threonine	324.20	6.12	38.61	2.53
Chromium	<3	Thorium	<0.03	C16:4 *n*-3	0.33 ± 0.01	Alanine	578.68	10.92	51.55	6.59
Caesium	<0.05	Thulium	<0.001	C18:0	3.96 ± 0.01	Proline	367.39	6.93	42.29	9.79
Copper	11.7 ± 1.17	Uranium	<0.04	C18:1 *n*-9	16.49 ± 0.02	Tyrosine	92.43	1.74	16.74	0.03
Dysprosium	<0.009	Vanadium	<0.75	C18:1 *n*-7	2.66 ± 0.06	Valine	175.77	3.32	20.59	2.94
Erbium	<0.005	Tungsten	<0.1	C18:2 *n*-6	4.39 ± 0.01	Methionine	106.07	2.00	15.82	0.03
Europium	<0.004	Yttrium	<0.15	C18:3 *n*-4	2.77 ± 0.01	Isoleucine	162.29	3.06	21.28	2.57
Gallium	<0.16	Ytterbium	<0.004	C18:3 *n*-3	1.45 ± 0.01	Leucine	280.66	5.29	36.81	4.64
Gadolinium	<0.01	Zinc	266.3 ± 26.63	C20:1 *n*-9	0.93 ± 0.01	Phenylalanine	144.35	2.72	23.84	2.51
Germanium	<0.1	Zirconium	<1	C20:1 *n*-7	0.69 ± 0.01	Lysine	266.45	5.03	38.95	5.66
Hafnium	<0.026	Silicon	<0.02	C20:2 *n*-6	2.11 ± 0.10	Hydroxyproline	-	-	-	7.51
Holmium	<0.002	Aluminium	<0.02	C20:4 *n*-6	1.50 ± 0.01	Aspartic acid	-	-	-	7.29
Indium	<0.07	Iron	0.0021 ± 0.0003	C20:3 *n*-3	0.96 ± 0.01					
Lanthanum	<0.06	Manganese	0.00007 ± 0.000007	C20:5 *n*-3 EPA	10.53 ± 0.02					
Lutetium	<0.002	Magnesium	0.0092 ± 0.0009	C21:5 *n*-3	1.06 ± 0.01					
Molybdenum	<0.3	Calcium	0.047 ± 0.007	C22:4 *n*-6	0.30 ± 0.01					
Niobium	<0.06	Sodium	0.077 ± 0.011	C22:5 *n*-6	0.33 ± 0.01					
Neodymium	<0.04	Potassium	0.025 ± 0.0042	C22:5 *n*-3	3.10 ± 0.01					
Nickel	<4	Thallium	<0.03	C22:6 *n*-3 DHA	20.34 ± 0.01					
Lead	<0.6	Phosphorus	0.132 ± 0.013							
Praseodymium	<0.01	-	-							

* Amino acid composition of marine collagen peptide from salmon skin [38].

The fatty acid composition, which depends strongly on the diet of the salmon and the seasons, showed a high content of PUFAs especially, EPA and DHA, with 10.53% and 20.34%, respectively. Oleic acid (C18:1 *n*-9) and palmitic acid (C16:0) were also present in large amounts in salmon head lipids, with 16.49% and 17.04%, respectively (Table 1). The salmon head PUFA’s fraction accounted for 49.6% against 25% of saturated and monounsaturated fatty acids.

#### 2.1.2. Characterization of Proteins

Protein identification was performed using the National Center for Biotechnology Information (NCBI) non-redundant-Chordata database by the combination of MASCOT and Peaks DB search programs for identification, characterization and quantitation of proteins. Taxonomy of the identified proteins does not necessarily correspond to salmon (*Salmo salar*) but to the other fish species containing homologous proteins with a slight difference (Table 2). The fast myotomal muscle actin is found in salmon head and in other fish like rainbow trout (*Oncorhynchusmykiss*) and red sea bream (*Pagrus major*) [39]. It has been reported that marine collagen peptides protect brain against oxidative stress [38].

The results of amino-acid composition (Table 1) showed that proteins of salmon head’s phospholipopeptidic complex (PLPC) were rich in glycine > glutamic acid > asparagines > alanine. We identified six essential amino acids among the nine: leucine, lysine, valine, isoleucine, phenylalanine and methionine. The percentage of the essential amino acids varied from 5% of leucine to 2% of methionine. It has been reported that histidine, proline, tyrosine and lysine have an antioxidant effect [40]. On the other hand, salmon head proteins contain 5% of lysine and 1.7% of tyrosine. The study of Li-Chan *et al.* [41], has demonstrated that salmon skin gelatin (*Salmo salar*) contained a high amount of glycine and low concentration of histidine. These results are in accordance with those obtained by Pei *et al*. [38], and our findings. The high content of branched and aromatic amino acids like Ile, Val, Phe and Tyr ensure an anti-hypertensive potential [33].

**Table 2:** Identification of native protein from peptidic fraction of the phospholipopeptidic complex.

Protein groups	Parent protein	Score (%)	*m*/*z*
1	Collagen 1a1 (Oncorhynchusmykiss)	99.21	137,117
2	Fast myotomal muscle actin (Salmosalar)	99.17	41,932
3	Collagen alpha-2(I) chain precursor (Oncorhynchusmykiss)	99.14	126,985
4	Type I collagen alpha 2 chain (Oncorhynchusketa)	99.13	126,443
5	Myosin heavy chain (Oncorhynchusketa)	99.05	222,153
6	Creatine kinase-2 (Salmosalar)	98.87	39,470
7	Collagen a3(I) (Oncorhynchusmykiss)	98.84	137,758
8	Collagen 1a (Salmosalar)	98.69	35,016
9	PREDICTED: Sarcoplasmic/Endoplasmic reticulum calcium ATPase 1-like isoform 2 (Ailuropodamelanoleuca)	98.47	109,208
10	Glyceraldehyde phosphate dehydrogenase (Oncorhynchusmykiss)	97.89	7,035,481
11	Myosin regulatory light chain 2 (Salmosalar)	97.79	18,996
12	Glycogen phosphorylase muscle form (Salmosalar)	97.67	97,485
13	Nucleoside diphosphate kinase A (Salmosalar)	84.25	17,092
14	Troponin C skeletal muscle (Salmosalar)	81.75	18,239
15	Enolase 3-1 (Salmosalar)	81.6	47,246
16	Hemoglobin subunit beta (Salmosalar)	70.85	16,124
17	Parvalbumin beta-1 (Salmosalar)	66.48	11,901
18	Unnamed protein product (Oikopleuradioica)	65.23	140,061
19	Histone cluster 2 H2ab (Daniorerio)	59.52	13,631
20	Hemoglobin subunit beta-1 (Salmosalar)	61.73	16,016
21	Unnamed protein product (Tetraodonnigroviridis)	61.63	351,956
22	Adenylate kinase (Salmosalar)	61.61	21,288
23	Myosin light polypeptide 3-1 (Salmosalar)	58.05	21,001
24	Alpha-globin (Salmosalar)	55.94	15,142
25	Collagen type I alpha 3 chain (Dicentrarchuslabrax)	51.66	137,004
26	l-Lactate dehydrogenase A chain (Salmosalar)	50.9	36,327

#### 2.1.3. Minerals and Element Trace Composition

After enzymatic digestion, the obtained phospholipopeptidic complex is composed of the insoluble peptide and lipids. PLPC was particularly enriched in zinc (266.3 μg/g), strontium (34.21 μg/g) and copper (11.7 μg/g) (Table 1). The study of Liaset and Espe [42], has shown the presence of zinc as a major component in salmon insoluble peptides.

Generally, marine organisms are an important source of minerals derived from the diet. Aubourg *et al.* [43], have reported from several studies that trace minerals’ concentrations in fish are not only influenced by the environment and food sources but also by anatomical and physiological aspects. The objective of the minerals investigation was to check the presence of toxic heavy metals such as mercury, cadmium, lead and chromium. These results showed no presence of toxic heavy metals.

#### 2.1.4. Treatment Characterization

**Figure 1:**
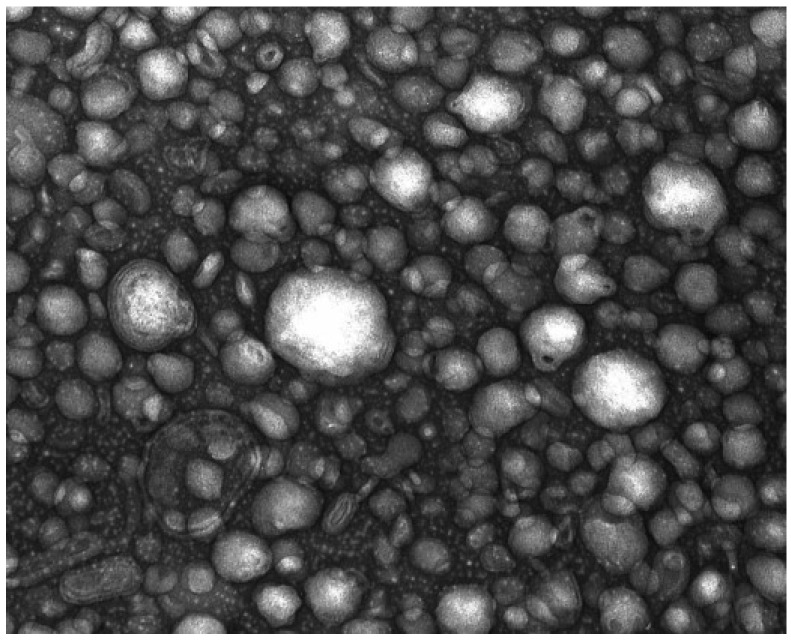
Transmission electron microscopy image of lipidic fraction mixed with distilled water.

The particle size distribution of peptidic powder was between 16 and 59 μm (data not shown). The TEM images revealed the presence of nanoliposome and nanoemulsion droplets. Lipidic fraction of PLPC contains about 35% of salmon oil which leads to the formation of nanoemulsion droplets and nanoliposomes stabilized by the phospholipids from the polar fraction (65% of polar lipid from PLPC). We observed homogenous spherical nanodroplets with good integrity (Figure 1). An electron microscopy tool should inevitably be associated to light scattering techniques to characterize and then to evaluate the stability of nanodroplets [44]. The mean particle size measured immediately after the sonication step was about 109 ± 2 nm, with a large polydispersity index (0.38 ± 0.01). Because of the negative charges of phosphatidylserine and phosphatidylinositol, electrophoretic mobility of nanoparticles was −3.9 ± 0.1 μm·cm/V.s. Electrophoretic mobility was determined under an electric field to characterize the surface charge of nanoparticles. The mean particle size and electrophoretic mobility were stable for 30 days at 25 °C. On the other hand, salmon head lipids were stable against oxidation phenomena for 30 days of storage under air ambiance at 30 °C [45].

### 2.2. *In Vivo* Test

#### 2.2.1. Body Weight

At the beginning of the experiment (D0), weights were equivalent across all mice groups. Significant decreases in body weights were observed at D4, in both peptidic and lipidic double dose (DD) treated mice when compared to controls (*p* < 0.05). Nevertheless, from D8 until the end of the treatment duration, no significant differences were found between groups (data not shown).

#### 2.2.2. Behavioral Test

##### 2.2.2.1. Elevated plus Maze (EPM)

**Figure 2:**
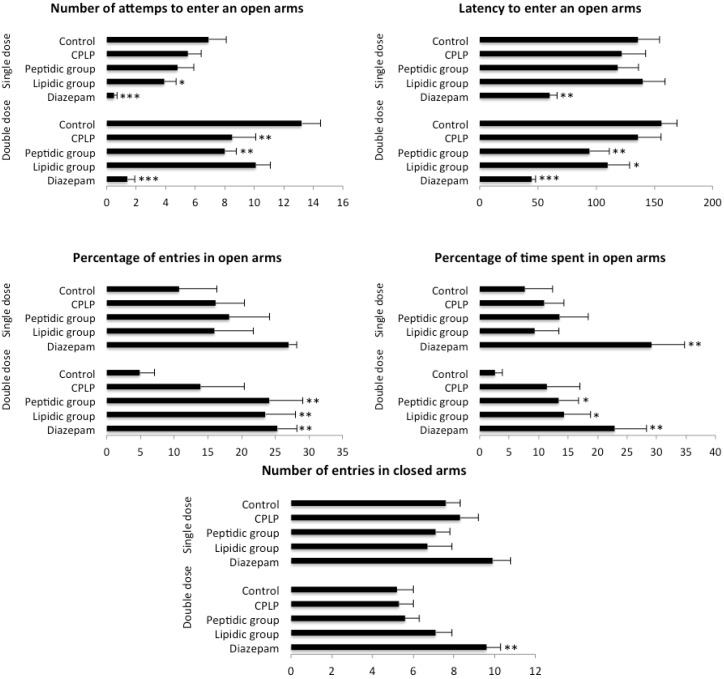
Results of elevated plus maze (EPM) test (* *p* < 0.05, ** *p* < 0.01, *** *p* < 0.001).

The effects of PLPC as well as its peptidic and lipidic fractions on mice behavior in the elevated plus maze are shown in Figure 2. Whatever the treatment, no effect was revealed in the EPM test following an acute single dose (SD) administration. However, regarding anxiety-related variables in the DD groups, a clear anxiolytic-like effect was found for the peptidic and the lipidic fractions of the PLPC. A significant increase in the percentage of open arm entries (*p* < 0.01) and time spent on these unprotected arms (*p* < 0.05) were observed in animals treated with the peptidic fraction of the complex. Moreover, those results were accompanied by a significant reduction of the attempts to enter the open arms (*p* < 0.01) and the latency of the first entry on an open arm (*p* < 0.01).

Animals treated with the lipidic fraction showed a similar effect, but not an as pronounced effect as the peptidic fraction. The percentage of open arm entries and the percentage of time spent on open arms increased significantly compared to control animals (*p* < 0.01, *p* < 0.05, respectively) and the latency of first exit was also found to be significantly reduced (*p* < 0.05). Although EPM variables of PLPC treated mice have evolved in the direction of an anxiolysis, their values, however, did not reach statistical significance, except for the number of attempts that was found significantly diminished as compared to control (*p* < 0.01). No significant differences were observed among SD and DD groups regarding the number of entries into closed arms of the EPM, indicating an *a priori* absence of treatment effect on mice locomotor activity.

##### 2.2.2.2. Hole-Board Test

**Figure 3:**
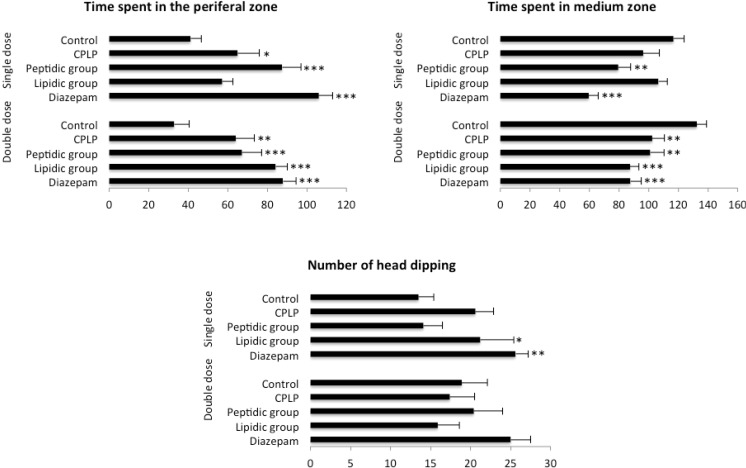
Results of hole-board test (* *p* < 0.05, ** *p* < 0.01, *** *p* < 0.001).

The hole-board test was conducted after one week of daily treatment. Results obtained in this test are shown in Figure 3.

Animals treated with PLPC at SD where found spending more time in the peripheral part of the board when compared to control (*p* < 0.05), whereas *post*-*hoc* Fisher’s Protected Least Significant Difference (PLSD) test revealed a close to significant result in the number of head dipping performed by PLPC-treated mice (*p* = 0.07), suggesting a slight anxiolytic effect of the complex.

This effect was more pronounced with the peptidic fraction, since mice treated at SD with salmon head peptides spent significantly more time in the peripheral zone (*p* < 0.001) in conjunction with a reduced time in the medium zone of the hole board compared to control (*p* < 0.01). However, there was no incidence of this treatment in the number of head-dippings.

Albeit animals treated with the lipidic fraction of the PLPC at SD performed more head-dipping than controls (*p* = 0.052). Their time spent ambulating in the peripheral and medium part of the hole-board was found similar to that observed in control mice, indicating an absence of effect.

PLPC DD-treated animals exhibited a significantly greater time spent in the peripheral part of the apparatus compared to control (*p* < 0.01). This result was also observed in mice treated with the peptidic and lipidic fractions of the complex with *p* < 0.01 and *p* < 0.001, respectively. Concerning the time spent in the medium part of the hole-board, mice from all treated groups were found to exhibit a significantly reduced time when compared to control (PLPC and peptidic fraction: *p* < 0.01), values of mice treated with the lipidic fraction reaching those of the diazepam group (*p* < 0.001). Thus, a clear anxiolytic profile was revealed for all the treatments in the hole-board test, although due to a great dispersion of values among groups, there was no effect concerning the number of head-dippings.

##### 2.2.2.3. Light-Dark Box Test

**Figure 4:**
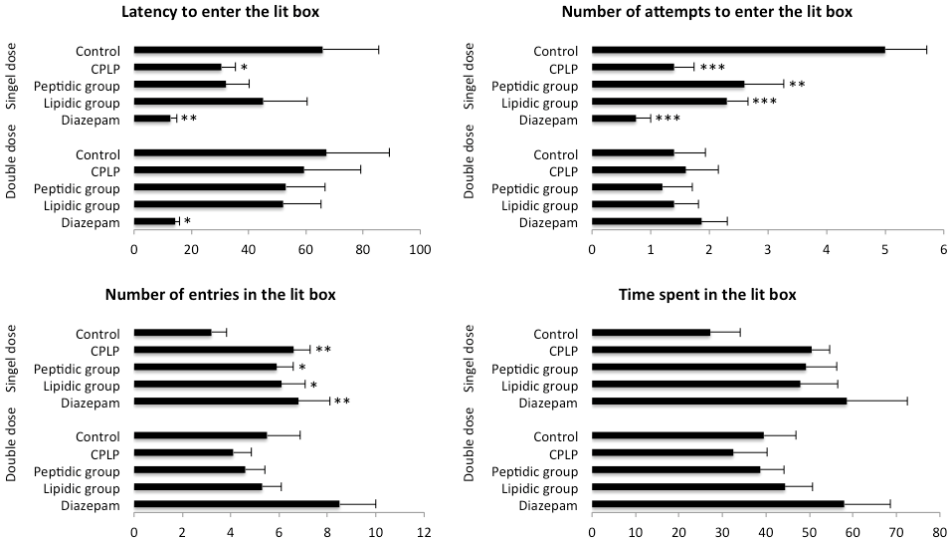
Results of light-dark boxtest (* *p* < 0.05, ** *p* < 0.01, *** *p* < 0.001).

Animals treated with the PLPC at SD for 14 days exhibited an anxiolytic-like profile in the light-dark box test. The number of attempts to move the lit box was found significantly reduced (*p* < 0.001) in conjunction with a significant increase in the number of entries to the lit box (*p* < 0.01) (Figure 4).

Assuming the close-to-significance ANOVA (*F* = 2.37; *p* = 0.067) obtained in the latency to enter in the lit box, the results for the PLPC group were also found in accordance with a reduced anxiety-like behavior toward the aversive area of the apparatus (*p* < 0.05), and the cumulative time spent in the lit box was found to be increased compared to control, but no clear conclusion can be made because of the high variability observed in this parameter.

Treatment of animals by both lipidic and peptidic groups showed a weaker anxiolitic-like response when treated at SD, exhibiting a significant reduction in the number of attempts to move into the lit box (*p* < 0.01; both groups), as well as a significant increase in the number of entries into the lit box (*p* < 0.05; both groups). Although, the total time spent in the lit box was found to increase in both groups compared with control animals. The comparison failed to reach significance due to a strong variability observed in whole tested groups, including diazepam.

Whatever the treatment, supplemented animals for 14 days at DD exhibited no anxiolitic-like activity in the light dark box test.

##### 2.2.2.4. Open Field Test

PLPC, as well as its peptidic or lipidic fractions showed no effect on the locomotor activity and on the vertical activity of mice assessed in the open field test, whatever the dose and the day of testing (D0, D7 and D14; result not shown).

### 2.3. ROS Evaluation in Neural Cells

At SD, there was no effect from the treatments on intracellular ROS content in neural cells, as revealed by the one-way ANOVA (Table 3). At DD, the peptidic fraction of the complex exhibited a scavenging activity (*p* < 0.01). Although, PLPC and its lipidic fraction seemed to induce a similar activity, the Fischer test *p* value for these groups did not reach significance compared to controls (*p* = 0.07 and *p* = 0.052, respectively).

**Table 3:** Effect of phospholipopeptidic complex and its peptidic and lipidic fractions on intracellular reactive oxygen species (ROS) accumulation in neural cells after 14 days of daily treatment. Results are expressed as mean ± SEM. ** *p* < 0.01 significantly different from control (Fisher *post hoc* test). The “t” indicated that the result approached significance.

Mean fluorescent intensity (arbitrary units)					ANOVA	
Dose	Control (*n* = 8)	PLPC (*n* = 8)	Peptidic fraction (*n* = 8)	Lipidic fraction (*n* = 8)	*F*_(3,28)_	*p*
SD	4.64 ± 0.68	3.72 ± 0.51	3.67 ± 0.30	4.76 ± 0.61	1.13	Not significant
DD	4.09 ± 0.41	3.43 ± 0.13 (t)	3.14 ± 0.11 **	3.48 ± 0.14 (t)	2.97	0.048

## 3. Discussion

The present study aimed to characterize the physico-chemical parameters of (i) A phospholipopeptidic complex obtained from enzymatic hydrolysis of salmon heads and composed of phospholipids, triacylglycerols, peptides and minerals; (ii) The peptidic fraction extracted from phospholipopeptidic complex; (iii) The lipidic fraction extracted from phospholipopeptidic complex. In addition, the evaluation and the monitoring of the anxiety status and locomotor activity were performed for two weeks in adult male mice—after supplementation with phospholipopeptidic complex—its lipid fraction and its peptide fraction.

Following the last behavioral tests, the assessment of treatments’ putative scavenging action was performed in mice neural cells after 14 days of dietary supplementation at single and double dose.

Concerning behavioral studies, the results obtained in the open field (OF) as well as in the elevated plus maze (EPM) (number of closed arms entries) indicated an absence of effect on the locomotor activity in mice whatever the treatment and the doses used. Consequently, the results obtained in the three anxiety-related tests based on rodent’s natural avoidance for aversive areas cannot be attributed to any stimulating or sedative properties of the treatments that would lead to false-positive results.

The total lipidic fraction of the complex was characterized and has been found containing about 50% of PUFAs, including about 20% of DHA and 10% of EPA. The incidence of PUFA (esterified on triacylglycerol) on anxiety is largely described in the literature [30,46,47]. In animal models, PUFAs-deficient mice exhibit greater anxiety-like behavior [48,49], especially when placed in an anxiogenic environment [50] or subjected to stressful conditions [51]. On the other hand, anxiety in PUFAs-deficient mice can be partly reversed with a chronic supplementation of phospholipids rich in *n*-3 PUFAs [48].

When looking to behavioral results obtained in mice treated with the complex’s lipidic fraction, no acute effect was found for the SD supplementation (36.9 mg/kg/day), as well as after a week of treatment. Nevertheless, when tested in the light-dark box test after 14 days of supplementation, mice exhibited a slight decrease of their anxiety level since their number of attempts to enter the lit box was found significantly reduced, in conjunction with an increased number of entries to the lit box. Although the total cumulated time spent in the lit box was found increased, statistical comparison with control animals did not reach significance. The authors explained this last result by a great variability in results among this variable.

Because the supplementation period was only sub-acute (14 days), we can hypothesize that the partial disinhibition of mice toward the aversive part of the light-dark box (LDB) should be seen as the first signs of the anxiolytic-like effects of PUFAs contained in the lipidic fraction. Indeed, previous works have demonstrated the positive impact of omega-3 fatty acids on anxiety via chronic supplementation in rats [52], non-human primates [53] and humans [30], but in this study, we did not show the effect of supplementation from longer periods.

Interestingly, an anxiolytic-like activity was revealed in the EPM for the DD (73.8 mg/kg) when acutely administered. This last result suggests firstly that the lipidic fraction induces acute anxiolytic-like effect, secondly that a quantitative threshold needs to be reached or exceeded for the activation of this activity, since no effect was found after a single SD administration (36.9 mg/kg), indicating that the induction of the anxiolytic-like activity of the lipidic fraction should be regarded as dose-dependent.

Chemical analyses have shown that 20% of this fraction was composed of DHA. Takeuchi *et al.* [49], have shown that these major PUFAs, which reduces several stress responses in rats, is very much supposed to act via a gamma-aminobutyric acid type A (GABA_A_) receptor-mediated mechanism.

However, the anxiolytic-effect of the DD was found lowered at D7 and absent at D14. Thus, the progressive disappearance of the acute anxiolytic-like activity of the DD when chronically administered suggests a hepatic adaptation to this diet. In rodent, weight is a very sensitive index of animal welfare and the hypothesis of a dispositional tolerance occurring with a sub-acute supplementation is reinforced by the recovery of mice to normal weight (D8) following the early decline observed at D4.

Regarding the scavenging effect of this fraction after two weeks of daily administration, the endogenous ROS content was found reduced in neural cells. This latter fact could be partly due to the high content of DHA found in the lipidic fraction, as several studies have shown that this predominant structural fatty acid of the central nervous system exerts an antioxidant action on brain lipids [54,55,56].

Enzymatic hydrolysis is a green method of peptide extraction from raw protein materials. When applied on a substrate such as salmon heads, it yields in a mixture of several peptides and amino-acids (Table 1 and Table 2). In anxiety paradigms, animals supplemented with the peptidic fraction of the complex showed behavioral responses that depended on the dose and duration of the treatment. Concerning the SD (86.1 mg/kg), no acute effect was found in the EPM consecutively to a single administration. Subsequently, the hole board test (HBT) and LDB tests revealed an anxiolytic-like effect after seven and 14 days of supplementation, respectively. When administered at DD (172.2 mg/kg), the effect of the PLPC peptidic fraction was found similar to that obtained with the lipidic fraction, *i.e.*, an acute anxiolytic-like effect that disappeared with the chronicity of the supplementation.

It is known that bioactive peptides are inactive or latent in parent protein, but are released in an active form by enzymatic hydrolysis. Applied on fish substrates, it allows for obtaining peptides showing antihypertensive [57] or antioxidant properties [58], or is linked to a relaxing effect [59]. Several recent reviews provide an overview about biological activities of marine peptides but did not identify any anxiolytic-like effect of such compounds [20,60,61]. It has been already shown that bioactive peptides from dairy products may exert an anti-anxiety activity [62], but to the best of our knowledge, it is the first time that a fish hydrolysate is studied and characterized for its anxiolytic-like properties.

According to the behavioral results obtained in this study, the peptidic fraction seems to have bimodal activity that is dose and time dependent.

As well as the lipidic fraction, the peptidic part of the complex was found to be able to induce an anxiolytic-like response when acutely administered at DD, unlike of the SD, suggesting a dose-dependent relationship.

It is known that protein breakdown is metabolic energy dependent. Although further experimentations are needed, because in DD-supplemented mice an animal’s weight was found to decrease after four days and return to normal values at D8, in conjunction with an absence of anxiolytic response at D7, we can hypothesize that the subacute administration of the DD induced a progressive hepatic tolerance. This fact can be attributed to the treatment chronicity, as well as the high quantity of compounds administered, leading to the elevation of liver enzymes, the degradation of compound occurring faster or at a greater scale, with the result of a decreased quantity of active compounds at receptors sites. Interestingly, no weight change was noticed in SD treated animal in parallel with, and the absence of, acute behavioral activity, and the implementation of an anxiolytic-like activity was obtained after a one week duration that extended even after two weeks of supplementation. Together, these observations, as well as results obtained in the DD, suggest that the absorption and bioavailability of the peptidic fraction differ according to the quantities administrated.

Following a 14-day supplementation, the level of ROS content in mice’ neural cells was found significantly decreased compared to control. It has been reported in literature that fish is an important source of antioxidant peptides obtained from enzymatic hydrolysis [20,63], low molecular weight peptides exhibiting a great potential to scavenge ROS *in vitro* [24,58]. The redox status of neural cells assessed *ex vivo* confirms that peptides obtained by enzymatic hydrolysis of salmon heads exert an efficient scavenging action at central level, conferring to the peptidic fraction a potential neuroprotective role.

The follow-up of anxiety in mice supplemented with PLPC at SD (123 mg/kg) revealed a progressive implementation of an anxiolytic-like activity, and this kinetic was found similar to that exhibited by the peptide fraction at SD. The lipidic fraction administered at SD was also found displaying an anxiolytic-like activity after two weeks of supplementation. The results obtained in PLPC mice at D14 did not showed any synergetic effect of both fractions in the complex.

Interestingly, mice treated with a single administration of PLPC at DD (246 mg/kg) did not show any behavioral change compared to controls in the EPM, this result being opposite to that observed with both peptidic and lipidic fractions. The lack of acute anxiolytic-like activity for the PLPC DD group revealed therefore an antagonism between the two fractions that are effective at DD when singly administered. It has been shown that bioactive peptides may be absorbed intact through the intestinal tract toward the circulatory system where they will produce their physiological effect, or may produce local effects [64]. Thereby, we suppose that the association between lipids and peptides as lipoprotein in the complex may decrease the bioavailability of bioactive compounds and consequently jeopardizes their effectiveness in anxiolysis. This hypothesis is also reinforced by the absence of body weight decrease in PLPC DD treated animals, unlike what was observed at D4 in animals treated with each DD single fraction.

Thus, there is a convergence of evidence that, according to the dosage, metabolism acts differentially with PLPC compounds, which exert an anxiolytic-like effect at low doses following a subacute supplementation, with this effect being antagonized at high doses.

The assessment of intracellular ROS contained in neural cells of PLPC DD-treated mice indicates a statistical trend in favor of a decrease of ROS. This close-to-significance result reveals a probable antioxidant capacity for the complex, which may be dose dependent even though no effect was found for SD supplemented mice. However, in *in vivo* conditions, the antioxidant effect depends essentially on the bioavailability and the formulation of bioactive molecules.

## 4. Experimental Section

### 4.1. Materials

Phospholipopeptidic complex (PLPC) was produced in our laboratory by enzymatic extraction from salmon head (*Salmo salar*) without any organic solvent at low temperature [65]. Boron fluoride-methanol (BF_3_/methanol) and PUFA standards were provided from Sigma Aldrich (Lyon, France). Cyclohexane, diethyl ether, chloroform, toluene, hexane, acetonitrile and methanol were provided from Fisher (Strasbourg, France). All solvents used were HPLC grade.

### 4.2. Phospholipopeptidic Complex Characterization

#### 4.2.1. Minerals and Element Trace Composition

Minerals and elements trace composition were determined after combustion at 980 °C according to the method of [66]. Briefly, 300 mg of PLPC was fused with 900 mg of ultra-pure LiBO_2_ in an automatic tunnel oven for 60 min. The fusion glass was dissolved after cooling at room temperature in a mixture of HNO_3_ (1 mol·L^−1^), H_2_O_2_ (~0.5% v/v) and glycerol (~10% v/v) to obtain a dilution factor of 333. The PLPC sample was analysed by inductively coupled plasma-mass spectrometry (ICP-MS) (Sciex Perkin Elmer ELAN 5000a).

#### 4.2.2. Lipid Classes

The lipid classes of salmon heads lipids from phospholipopeptidic complex were determined by thin layer chromatography-flame ionization detector (Iatroscan MK-5, Iatron Laboratories Inc., Tokyo, Japan) [45]. The first migration step in hexane, diethyl ether and formic acid (80:20:0.2, v:v:v), was carried out to determine the percentage of neutral and polar lipid fractions. The second migration in chloroform, methanol and ammoniac (65:35:5, v:v:v), defines the composition and percentage of polar lipid’s classes. All standards were purchased from Sigma (Sigma-Aldrich Chemie GmbH, Munich, Germany). Area percentages were expressed as the mean value of five repetitions.

#### 4.2.3. Fatty Acid Composition

Fatty acid methyl esters (FAMEs) from salmon PLPC were prepared according to the Morrison & Smith’s method [67]. The transmethylation was performed using 1 mL of BF_3_ in methanol (14%, w/v) and 1 mL of toluene at 100 °C. After the extraction of FAMEs with cyclohexane, they were washed with distilled water and analyzed with a split mode by gas chromatography (CG-2010 Plus, Shimadzu, Kyoto, Japan) equipped with a flame ionization detector and a capillary column (60 m, 0.25 mm i.d. × 0.20 μm film thicknesses). Oven temperature was set at 200 °C; detector and injector temperatures were at 250 °C. Helium was the carrier gas at a flow rate of 0.79 mL·min^−1^. A temperature program of column was initially set at 120 °C for 2 min, then rose to 180 °C for 2 min at a rate of 2 °C/min and kept at 220 °C for 25 min. FAMEs (PUFA1 from marine source and PUFA2 from vegetable source; Supelco, Sigma-Aldrich, Bellefonte, PA, USA) were used as standards to identify fatty acids. The percentage of FAMEs was calculated from the total area of all peaks. The results were presented as triplicate analyses.

#### 4.2.4. Determination of Amino Acid Composition

According to Gbogouri *et al.* [32], the freeze dried sample was hydrolysed in 6M HCl solution with phenol (1%) at 150 °C for 60 min under vacuum, in Pico-Tag system (Waters, Milford, MA, USA). Then, the high performance liquid chromatography equipped with a PTC RP-18 column (2.1 mm × 22 cm) was used to separate the phenylisothiocyanate amino acid derivatives (Applied biosystems model 172A, Applera Corp, Foster City, CA, USA). Two buffers were used to elute the amino acids derivatives: Sodium acetate (45 mM, pH 5.9) and a mixture of sodium acetate (30%, 105 mM, pH 4.6) with acetonitrile (70%). The detection was made at 254 nm with an Applied Biosystems detector (model 785A Applera Corp, Foster City, CA, USA).

#### 4.2.5. Proteomics

The identification of proteins was carried out using nano-LC LTQ Orbitrap XL (NanoLC Ultimate 3000, Dionex, Amsterdam, The Netherlands) coupled with LTQ Orbitrap XL hybrid mass spectrometers (Thermo Scientific, Courtaboeuf, France). An aliquot of PLPC (10 mg) was solubilised in 1 mL of ultrapure water (Milli-Q, Millipore, Guyancourt, France). After 15 min incubation at room temperature under agitation, the sample was centrifuged at 15,000× *g* for 10 min. The supernatant’s peptide/protein was separated using a C18 pre-column (5 μm, 300 Å/300 μm i.d. × 5 mm) and C18 column (3 μm, 100 Å/75 μm i.d. × 150 mm) and then analyzed by proteome Discoverer 1.2 software (Thermo Scientific). Solvent A was composed of water and formic acid mixture (100/0.05 v/v). Solvent B was composed of acetonitrile and formic acid mixture (100/0.05 v/v). The flow rate and UV detection were set at 250 nL/min and 214 nm, respectively. The gradients were as follows: 140 min LC method at 30 °C; 60 min (2% A/25% B); 60–85 min (25% A/50% B); 85–105 min (5% A/90% B); 105–120 min (90% B); 120–127 min (90% A/2% B); 127–140 min (2% B). The identification of protein sequences was performed by the National Center for Biotechnology Information non-redundant (NCBInr) database using Mascot program (2.2.07, Matrix Sciences, Boston, MA, USA) and Peaks Studio software (4.5 BSI).

#### 4.2.6. Treatments and Doses

Different national health organizations recommended approximately 300–600 mg per day of EPA and DHA. In 2009, the German organization recommended 300 mg/day of EPA and DHA [68], while the French agency recommended 500 mg in 2010 [69]. The phospholipopeptidic complex is used as a dietary supplement of EPA and DHA.

In this study, the single dose of phospholipopeptidic complex was set at 123 mg/kg according to the mice-human dose conversion from FDA [70]. The double dose was used in order to assess the effect of a strong supplementation.

The phospholipopeptidic complex is composed of 30% of lipids and 70% of hydrolyzed proteins. In this study, we tested the effect of the entire complex as well as its lipidic and peptidic fractions taken separately (Table 4).

**Table 4:** Quantities administered to mice.

Treatment	Single dose (SD)	Double dose (DD)
Phospholipopeptidic complex	123 mg/kg/10 mL	246 mg/kg/10 mL
Lipidic fraction of the PLPC (30%)	36.9 mg/kg/10 mL	73.8 mg/kg/10 mL
Peptidic fraction of the PLPC (70%)	86.1 mg/kg/10 mL	172.2 mg/kg/10 mL

Lipidic and peptidic fractions were separated by the method of Folch *et al.* [71]. Total PLPC and peptidic fractions were dispersed in distilled water under magnetic stirring before each feeding. However, the lipidic fraction was dispersed in distilled water, vortexed for 30 min and sonicated at 40 kHz at 40% of full power for 120 s (1 s on and 1 s off) to obtain homogenous nanodroplets solution according to Belhaj *et al.* [72].

Mice received a daily oral administration (*per os*) over a 14-day period. On days when animals were tested, treatments were administrated one hour before testing except for the reference group (30 min previously to test).

##### 4.2.6.1. Static Light Scattering

The static light scattering was used in order to determine the size of PLPC particles. 0.5 mg of PLPC was dispersed in 100 mL of prefiltered distilled water (Millipore France, Molsheim, France; membrane diameter 0.22 μm). The measurement was carried out under stirring conditions at 2000 rpm. The average diameter of five measurements was calculated from the Mie theory.

##### 4.2.6.2. Transmission Electronmicroscopy (TEM)

The structure of nanoliposomes was observed using a negative staining method [73]. The sample was diluted 10-folds with distilled water and mixed with 2% of ammonium molybdate solution at the same volume. After 3 min at room temperature, and 5 min on a copper mesh, the sample was examined using a Philips CM20 Transmission Electron Microscope operating at 200 kV linked to an Olympus TEM CCD camera.

##### 4.2.6.3. Particle Size, Electrophoretic Mobility and Polydispersity Index of Nanoparticles

The hydraulic diameter, electrophoretic mobility and polydispersity index of the elaborated nanodroplets were determined by dynamic light scattering using a Malvern Zetasizer Nano ZS (Malvern Instruments, UK) [45]. Samples were diluted (1:400) in ultra-filtrate distilled water. Refractive index (RI) and absorbance were respectively set at 1.471 and 0.01 at 25 °C. The average of three measurements was performed.

### 4.3. *In Vivo* Study

#### 4.3.1. Animals

96 Swiss albino male mice aged nine weeks (35–45 g) were obtained from Charles River (L’Arbresle, France). Upon arrival, they were housed four mice per cage (one mouse per treatment and one control) and kept under a standard 12 h-light/12 h-dark cycle (lights on at 8 pm, lights off at 8 am) in a controlled temperature room (22 ± 2 °C). Foodpellets (SDS Dietex, Argenteuil, France) and tape water were provided *ad libitum*. Mice were weighed every four days to allow the exact determination of the treatment volume (10 mL/kg b.w.) and to report possible weight change due to treatment. All these experiments on animals were performed in respect to the rules provided by the European Union Directive [74]. The animal care unit is authorized by the French Ministries of Agriculture and Research (Government Authorization No. 57-463-2).

#### 4.3.2. Study Design

Two separate studies following the same experimental design were performed for behavioral and free radicals scavenging evaluations. Each study involved the three treatments and control; the treatment doses were doubled in the second study*.*

Treatments were administered daily for two weeks. The follow up of the anxiety status began on the first day of administration (D0, acute effect), followed by a new assessment each seven days (D7, D14). To avoid habituation to a single experimental paradigm, three different tests were conducted: The elevated plus maze test (EPM) on D0, the hole board test (HB) on D7 and the light-dark box test (LDB) on D14 [75,76,77,78]. A reference group of eight mice orally treated with diazepam at the anxiolytic dose of 1.5 mg/kg b.w was added for each behavioral test. Moreover, an open field test was performed 15 min after each anxiety test to assess the mice locomotor activity.

#### 4.3.3. Behavioral Tests

##### 4.3.3.1. Elevated Plus Maze (EPM)

On D0, mice were tested in the elevated maze. The apparatus is made in dark polyvinyl plastic and consist of a maze elevated to a height of 50 cm with two open (30 × 5 cm, 400 lux) and two closed arms having walls (30 × 5 × 15 cm, 90 lux), arranged so that arms of the same type were opposite to each other, the four arms extending from a common central platform (5 cm × 5 cm). To prevent mice from falling of the open arms, a rim (2.5 mm high and 8 mm deep) surrounded the perimeter of the open arms. Testing took place from 10.00 am to 1 pm. The mouse was initially placed on the central platform of the apparatus, facing closed arms and left freely to explore it for 3 min. All test sessions were recorded by a video camera positioned above the apparatus.

The test procedure, adopted from Lister, measured the number of entries in the open and the closed arms, the total number of entries as well as the cumulated time spent in the open arm [77]. The results are expressed in percentage of entries into the open arms and percentage of time spent in the open arms. According to the general procedure, an arm entry was counted when the animal placed all four paws into the arm. The latency of the first entry into an open arm, as well as the number of attempts for an open arm entry (the mouse stretches forward and retracts to original position on the central platform) were also recorded. After each test, the apparatus was cleaned with a 10% ethanol solution.

##### 4.3.3.2. Hole Board Test (HBT)

The HB test was conducted on D7. The apparatus consisted of a grey square open area (45 × 45 cm placed 80 cm height) with 16 equally spaced holes (4 × 4, 2.5 cm diameter) in the floor, delimiting peripheral, medium and central areas. Testing took place from 10.00 am to 1 pm. The HB was illuminated with a very dim red light (40 lux) [79]. The mouse was initially placed in the center of the HB and allowed to freely explore the apparatus for 3 min. Test sessions were video tapped and the time spent in peripheral and medium areas as well as the number of head dips were recorded. A head dips was counted if both eyes disappeared into the hole. The open-field was wiped with a 10% ethanol solution after each trial and any faeces removed.

##### 4.3.3.3. Light-Dark Box Test (LDBT)

The apparatus consisted in a two compartments (light/dark, surface ratio 3:2) divided into 15 squares: 9 × 9 cm. The dark box (black PVC, 27 × 18 × 29 cm) is illuminated by a dim red light (50 lux) and is divided into six squares. The lit box (white PVC, 27 × 27 × 29 cm) is illuminated by an aversive white light (200 lux at the level of the floor) and is divided into nine squares. The two compartments are connected by an opening door (7 × 7 cm). Testing was performed from 10.00 a.m. to 1 p.m. At the beginning of the test, the mouse was placed in the light box with its head facing the door of the dark box and its behavior was video recorded for 3 min. The following parameters were monitored: the latency time of the first entry into the lit box, the number of attempts to enter into the lit box, the total number of entries into the lit box and the cumulative time spent in the aversive compartment. Between each trial the apparatus was cleaned with a 10% ethanol solution.

##### 4.3.3.4. Open Field Test (OF)

On days when the status of anxiety was evaluated, the general locomotor activity of mice was assessed in an adjacent room, using a circular OF. The apparatus floor, made of white polyvinyl plastic, is divided into 36 squares delineating peripheral, medium and central circular areas. The assessment was performed under red light (120 lux).

Mice were initially placed head facing the apparatus’ wall (grey PVC) and after 1 min of habituation, the number of squares crossed with the four paws (horizontal activity) and the number of rearings (vertical activity) were video recorded for 3 min by a camera fixed above the apparatus.

#### 4.3.4. ROS Evaluation in Neural Cells

On day 14, eight mice of each group were anaesthetized with halothane (Laboratoire Belamont, Paris, France) after the open field test and sacrificed by decapitation. The brain was collected and placed into a 1.5 mL tube containing 0.5 mL of cold phosphate buffered saline (PBS) and softly homogenized using an inoxmicropotter (Eppendorf, Le Pecq, France). Tissue digestion was performed at room temperature using 0.2% collagenase II (Invitrogen, Saint Aubin, France) for 30 min. After discarding supernatant, cells were re-suspended with buffer solution (1 × PBS 2 mL; EDTA 2 mM; bovine serum albumin (BSA) 0.5%) and filtered through a 70 μm nylon mesh (Miltenyi, Paris, France). The filtrates were collected and centrifuged (894× *g*, 10 min and 4 °C) and the supernatants were completely removed. After an addition of 2 mL of the buffer solution, samples were centrifuged again (894× *g*, 10 min and 4 °C) and the supernatants were completely removed. Cell pellets were re-suspended in 80 μL of buffer and 20 μL of anti-CD 90.2 Microbeads (Miltenyi) were added, allowing the selection of 10^7^ total neural cells. Samples were mixed and incubated during 15 min at 4–8 °C. Cells were then washed by 2 mL of buffer and centrifuged (894× *g*, 10 min and 4 °C). Supernatants were pipetted off entirely and cells were re-suspended in 500 μL of buffer.

We proceeded to the magnetic separation according to manufacturer’s instructions (Miltenyi Biotec, France). The eluted cells were centrifuged twice (10 min at 800× *g* and 4 °C), with removal of the supernatant at each centrifugation step. Neural cells were re-suspended in 0.5 mL of buffer solution for ROS measurement. To this aim, 5 μL of dichlorfluorescein-diacetate (50M) were added to each sample before being incubated at 37 °C for 15 min. In presence of peroxides, the oxidation of DCFH-DA leads to the highly fluorescent compound dichlorofluorescein (DCF) which results in an increase of fluorescence at 525 nm, when excited at 488 nm [13]. When the incubation was completed, samples were placed on ice for 5 min to stop the reaction and the ROS level was measured using a FACScan flow cytometer (Becton Dickinson, Rungis, France) via channel FL1 detection. Data were collected for 10,000 events.

#### 4.3.5. Data Analysis

Data distribution was preliminarily analyzed using the Skewness in conjunction with the statistical Kurtosis [80]. All behavioural data had a Gaussian distribution and were consequently analyzed by a one-way ANOVA followed by a *post*-*hoc* PLSD Fischer test. Results were reported as mean ± SEM. Since body weight was found having a non-gaussian distribution, it was consequently analyzed using the Kruskal-Wallis test. When the Kruskal-Wallis test lead to significant results, *post hoc* comparisons between controls and treated groups were performed using a Mann-Whitney *U*-test. Statistical analyses were carried out using Statview^®^ 4.5 software (Abacus Concepts, Inc., Berkeley, CA, USA). Significance was set at *p* < 0.05.

## 5. Conclusions

This study showed that a phospholipopeptidic complex obtained from salmon heads’ enzymatic hydrolysis, and its lipidic and peptidic fractions exert a dose and time dependent anxiolytic effect. At acute double doses, we obtained an anxiolytic-like effect that disappears with time supplementation. A dietary administration of salmon phospholipopeptidic complex—its lipidic and peptidic fractions administered separately at single dose—have an anxiolytic-like effect on mice during 14 days of treatment, regardless the treatment.

However, there is no correlation between daily supplementation of treatments and oxidative status of neural cells. A significant ROS diminution was observed at DD of lipidic and peptidic fractions of phospholipopeptidic complex separately.
